# Why do scientific advances take so long to be incorporated into clinical practice? The case of intracameral injection of antibiotics to prevent acute endophthalmitis after cataract surgery

**DOI:** 10.5935/0004-2749.2022-854

**Published:** 2025-08-21

**Authors:** Mathias Violante Melega, Monica Alves, Rodrigo Pessoa Cavalcanti Lira

**Affiliations:** 1 School of Medical Sciences, Universidade Estadual de Campinas, Campinas, SP, Brazil; 2 Universidade Federal de Pernambuco, Recife, PE, Brazil

In the last 20 years, several studies have confirmed the safety and efficacy of the intracameral injection of antibiotics to prevent acute endophthalmitis after cataract surgery^(1,2)^. In 2007, a major clinical study by the European Society of Cataract and Refractive Surgeons (ESCRS)^(3)^ demonstrated the benefits of intracameral cefuroxime. Recently, three Brazilian studies have demonstrated the safety and efficacy of intracameral moxifloxacin^(4-6)^. Nevertheless, the adoption of such scientifically-proven strategies in clinical practice is hindered by other factors. Perhaps the largest obstacle is the lack of commercially available intracameral antibiotics in various parts of the world, including Brazil.

Despite high quality and evidence-based research to support them, various treatment strategies are still considered off-label. These off-label uses can be incorporated into an ophthalmologists’ therapeutic toolkit. However, this depends on the accessibility of a given treatment. Examples include, the use of topical atropine to prevent the progression of myopia in children^(7)^, interferon alfa-2b to treat conjunctival and corneal neoplasia^(8)^, tacrolimus to treat allergic conjunctivitis^(9)^, and the intracameral injection of adrenaline to prevent or revert intraoperative miosis^(10)^. Although these strategies are scientifically supported, the lack of commercially available products or recognition by regulatory agencies results in their limited use in clinical ophthalmology practice.

Certainly, regulatory steps must be followed before a new drug or strategy can be safely produced and utilized. These steps generally comprise preclinical trials and subsequent three-phase clinical trials, followed by a review of the resulting evidence by the regulatory agencies responsible for licensing the production and sale of a given drug. As observed recently in the case of coronavirus drugs and vaccines, laboratory technical resources, scientifically rigorous clinical trials, and careful analyses of their results are essential for medical products to be authorized for use with target populations^(11)^.

Performing robust clinical trials that enable the authorization of a new drug is not an easy task. Such studies are expensive and agencies that invest in research must conduct cost-benefit analyses; however, the results are often unfavorable. The high degree of outcome uncertainty, the possible inability of a product to compete on the market, and insufficient projected profit border the reasons that hinder the process of new drug development and incorporation into the market.

Another obstacle to the adoption of evidence-based advances is the ethical considerations necessary for randomized clinical trials. If there is already sufficient clinical evidence to support a given treatment strategy, it is unethical to conduct further drug trials, either on humans or animals, regardless of whether the existing evidence is sufficient to satisfy regulating bodies.

The case of intracameral injection of antibiotics to prevent acute endophthalmitis after cataract surgery has generated controversy. The literature provides relevant scientific data to support the safety and efficacy of this treatment strategy. In fact, the European Medicines Agency (EMA) has already authorized the production and sale of Aprokam^®^, a drug specifically labeled for intracameral use in the European Union^(12)^. This product is in widespread use in European cataract surgeries and often replaces topical antibiotics in the postoperative period. Meanwhile, the same studies that supported the EMA’s decision were deemed insufficient by the Food and Drug Administration (FDA), the regulating body in the United States. The FDA cited certain limitations of the European study in their decision, an outcome that demonstrates how the interpretation of the same data by different agencies may impact the commercialization of new products.

A plethora of studies on new medical treatments are published every year; however, practical scientific advancement takes time and often never reaches patients. The complexity, financial cost, and risks involved in the process (from the initial studies in laboratories to production by the pharmaceutical industry) are the primary reasons why it is so difficult to put scientific findings into practice. Furthermore, the bureaucracy involved in regulatory agencies’ product assessments further slows the practical implementation of these scientific advances.

In the case of intracameral antibiotics in Brazil, collaboration between ophthalmologists, researchers, and the pharmaceutical industry is vital once the benefit from scientific advances in medicine are craved. Additional randomized clinical trials (and ideally multisite trials) would provide even more support for the production and sale of new drugs to prevent endophthalmitis after cataract surgery. Until this happens, ophthalmologists in Brazil must individually weigh the pros and cons of the scientifically supported but off-label use of intracameral antibiotics.
